# An Improved Self-Training Method for Positive Unlabeled Time Series Classification Using DTW Barycenter Averaging

**DOI:** 10.3390/s21217414

**Published:** 2021-11-08

**Authors:** Jing Li, Haowen Zhang, Yabo Dong, Tongbin Zuo, Duanqing Xu

**Affiliations:** College of Computer Science and Technology, Zhejiang University, Hangzhou 310027, China; zjujing@zju.edu.cn (J.L.); cszhw@zju.edu.cn (H.Z.); zuotongbin@zju.edu.cn (T.Z.); xdq@zju.edu.cn (D.X.)

**Keywords:** positive unlabeled time series classification, self-training, dynamic time warping, DTW barycenter averaging

## Abstract

Traditional supervised time series classification (TSC) tasks assume that all training data are labeled. However, in practice, manually labelling all unlabeled data could be very time-consuming and often requires the participation of skilled domain experts. In this paper, we concern with the positive unlabeled time series classification problem (*PUTSC*), which refers to automatically labelling the large unlabeled set *U* based on a small positive labeled set *PL*. The self-training (*ST*) is the most widely used method for solving the *PUTSC* problem and has attracted increased attention due to its simplicity and effectiveness. The existing *ST* methods simply employ the *one-nearest-neighbor* (*1NN)* formula to determine which unlabeled time-series should be labeled. Nevertheless, we note that the *1NN* formula might not be optimal for *PUTSC* tasks because it may be sensitive to the initial labeled data located near the boundary between the positive and negative classes. To overcome this issue, in this paper we propose an exploratory methodology called *ST-average*. Unlike conventional *ST*-based approaches, *ST-average* utilizes the average sequence calculated by DTW barycenter averaging technique to label the data. Compared with any individuals in *PL* set, the average sequence is more representative. Our proposal is insensitive to the initial labeled data and is more reliable than existing *ST*-based methods. Besides, we demonstrate that *ST-average* can naturally be implemented along with many existing techniques used in original *ST*. Experimental results on public datasets show that *ST-average* performs better than related popular methods.

## 1. Introduction

With the rapid development of the Internet of Things technology, a large number of time series generated by sensor devices have appeared in various fields, including PM2.5 sensing systems [1], activity tracking [2], real-time patient-specific ECG classification [3], and many more. Ubiquitous time-series have received a substantial research interest in clustering, classification, querying, prediction, and indexing of such data. Time-series classification (TSC) is the most widely used among all techniques applied to time-series data and has always attracted great attention [4].

**Figure 1:**
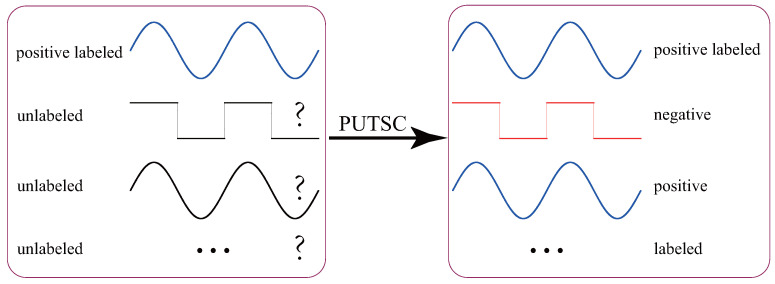
A simple example of *PUTSC*. Here we assume that the size of the *PL* set |PL|=1. The task of *PUTSC* is to automatically label the large set of unlabeled time-series *U* based on *PL* set.

Conventional supervised TSC tasks assume that all training data are labeled, and they train a high-quality classification model based on these labeled data. However, in practice, manually collecting and labelling all unlabeled training data could be very time-consuming and often requires the participation of skilled domain experts. On the other hand, with the popularity of various cheap sensors and the increase in storage capacity, a large number of unlabeled time-series data are being generated every day. Thus, the paradigm of supervised TSC is not practical in many real-life applications. Rather, the TSC tasks in real-life often involve *positive unlabeled TSC (PUTSC)* [5,6], which we study in this paper. Suppose that *PL* is a set containing only a small number of positive labeled time-series and *U* is a large set of unlabeled (positive and negative) time-series, the task of *PUTSC* is to automatically label the *U* set based on *PL* set. Figure 1 shows an example of *PUTSC*.

The *PUTSC* is a proper solution in the time-series data mining community when unlabeled time-series are easy to obtain but labelling them is difficult. In the past few years, numerous inspiring algorithms have been proposed to solve the *PUTSC* problem. The self-training based method (*ST*) [7] is the most popular *PUTSC* method and has been successfully used [5,8,9,10,11,12,13,14]. In this paper, we will particularly focus on *ST* technique due to its effectiveness and popularity. The idea behind *ST* method is straightforward: if an unlabeled time-series in *U* is very similar to a labeled time-series in *PL*, then this unlabeled one has a very high probability of being positive. Based on this idea, the *ST* labels the most similar time-series in *U* set to *PL* set as a positive one and adds this time-series to the *PL*. The process continues until a certain *stopping criterion* is met. The detailed description and illustration of the *ST* algorithm can be found in Section 2.

**Figure 2:**
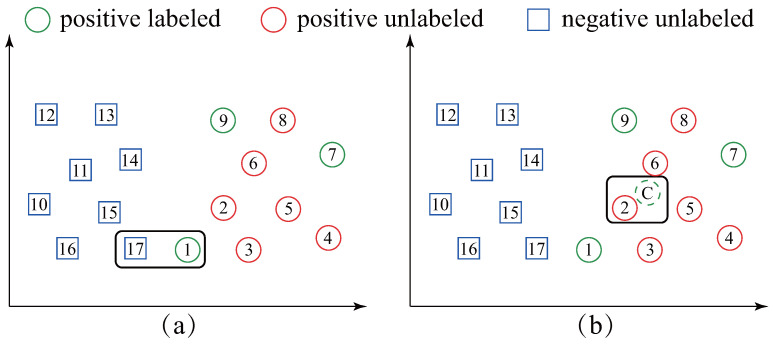
An illustration of the difference between *ST* based methods (**a**) and our *ST-average* method (**b**). *ST* labels the unlabeled data (square-17) which is closest to *PL* set while *ST-average* labels the unlabeled data (circle-2) which is closest to the average sequence of the *PL* set.

We note that all the *ST* based methods simply employ the *one-nearest-neighbor (1NN)* formula to determine which unlabeled time-series should be labeled and added to *PL* set. The *1NN* formula is straightforward; however, it is challenging to explicitly recognize the class boundary and may be sensitive to the initial labeled time-series located near the boundary between the positive and negative classes. For example, in Figure 2a, the *PL* set has three initial positive labeled data (circle-1, 7 and 9). The conventional *ST* based methods will label square-17 as the positive one and add it to *PL* because it is the most similar data in *U* to *PL*. Nevertheless, it is obvious that square-17 is negative data; thus, it leads to an incorrect result. More seriously, a chain effect of wrongs will follow after adding square-17 to the *PL* set (the square-15 will be added to *PL* because it is now the most similar data in *U* to *PL*). Surprisingly, despite this extensive literature, we are not aware of any work overcoming the drawback presented in Figure 2a.

Facing the aforementioned drawback, inspired by [15] which uses the *centroid* of time series to improve TSC efficiency and accuracy, in this paper, we propose an exploratory methodology called *self-training based on the average sequence of the time-series (ST-average)*. Our proposal, *ST-average*, is different from all the *ST* based works in that: *ST-average* labels the time-series in *U* which is the most similar to the *average sequence* of the *PL* set as a positive data. Figure 2b illustrates our idea. *ST-average* first calculates the average sequence of the *PL* set (circle-C in Figure 2b). Then, circle-2 is labeled as a positive one and added to *PL* set because it is the most similar time-series in *U* to circle-C. It can be seen from Figure 2b that our method is largely insensitive to the circle-1. Therefore, it is much more reliable than existing *ST* methods.

**Figure 3:**
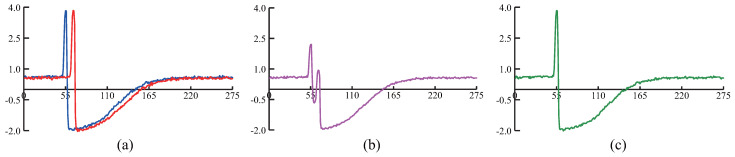
(**a**) two shifted time-series collected from the UCR archive. (**b**) the resulting average sequence when averaged under the Euclidean Distance. (**c**) the resulting average sequence when averaged under the DTW distance using DBA technique.

The *ST-average* is an apparent solution to *PUTSC* problem. Unfortunately, it is a challenging task to define the average sequence of the time-series. The simplest way is to compute mean value of all time-series using Euclidean Distance. However, this naive method is not feasible because time-series data are always shifted in time axis. Besides, this point-by-point averaging approach cannot capture actual shape of two time-series. For instance, Figure 3a presents two shifted time-series collected from the UCR archive [16]. Anyone could confirm that they are very similar to each other although they appear dephased in time. However, if we use Euclidean Distance averaging, the resultant undesired average sequence shown in Figure 3b will resemble none of the parent time-series. To obtain a reasonable average sequence, we compute average sequence of the time-series under the Dynamic Time Warping (DTW) [17] distance because DTW allows time-series to be locally shifted and stretched. Specifically, in this paper, we adopt a well-known technique, DTW barycenter averaging (DBA) [18], to compute average sequence of the time-series. Figure 3c shows the resultant average sequence when DBA is applied. Obviously, the result is correct and more natural when averaged using DBA technique.

To summarize, we make the following contributions.

- We point out that traditional *ST*-based methods may be sensitive to the initial labeled time-series located near the boundary between the positive and negative classes. To overcome this issue, we propose a novel method *ST-average* to solve the *PUTSC* problem by using the average sequence of the *PL* set to decide which unlabeled time-series should be labeled and added into *PL* set.
- It is not a trivial task to calculate the average sequence of the time-series set and we demonstrate the necessity of using DBA through experiments.
- The *ST-average* method is orthogonal to some of the stopping criteria and similarity measures used in *ST*-based methods. We show how *ST-average* can naturally be implemented along with them and present an explicit implementation of *ST-average*.
- We conduct experiments using public well-know time-series datasets to evaluate the performance of our proposal. Experimental results demonstrate that our method performs better than related competitors.

Our paper is organized as follows. Section 2 introduces the necessary background knowledge and related work of our research. Particularly, we focus on describing self-training technique for solving *PUTSC* problem. Our proposed method can be found in Section 3. Section 4 presents the experimental results on real-world datasets. The effectiveness and efficiency of the proposed method are reported in this part. We finally conclude this paper in Section 5.

## 2. Background and Related Work

Before formally elucidating the proposed *ST-average* algorithm, this section provides a brief overview of the background and related work.

### 2.1. Positive Unlabeled Time Series Classification

The deficit of labeled time-series data in the time-series data mining domain has motivated increasing research in the *positive unlabeled TSC (PUTSC)* topics. Given a *PL* set which contains only a small number of positive labeled time-series and a *U* set which is a large set of unlabeled (positive and negative) time-series, the task of *PUTSC* is to automatically label the *U* set based on *PL* set. For example, the *PUTSC* can be applied to classify ECG heartbeats as abnormal or normal based on some labeled abnormal ones.

The *PUTSC* can be regarded as a special case of semi-supervised learning (SSL) [19,20]. However, most existing SSL approaches cannot be used in *PUTSC* due to the specific characteristics of time-series data such as high dimension, noisy, different lengths and high feature correlation. In the specialized literature, as far as we know, only two main approaches have been proposed to tackle the *PUTSC* problem effectively. The first one is based on the clustering technique [6,21]. Representative methods include Learning from Common Local Clusters (LCLC) [6] and Ensemble based Learning from Common Local Clusters (En-LCLC) [21]. The second one is based on self-training technique [5,8,9,10,11,12,13,14]. We note that the majority of related works solving the *PUTSC* problem have used the self-training approach because its instance-based classifier best suits the particular features of time-series. Thus, in this paper, we follow this tendency.

### 2.2. Self-Training Technique for the PUTSC

**Figure 4:**
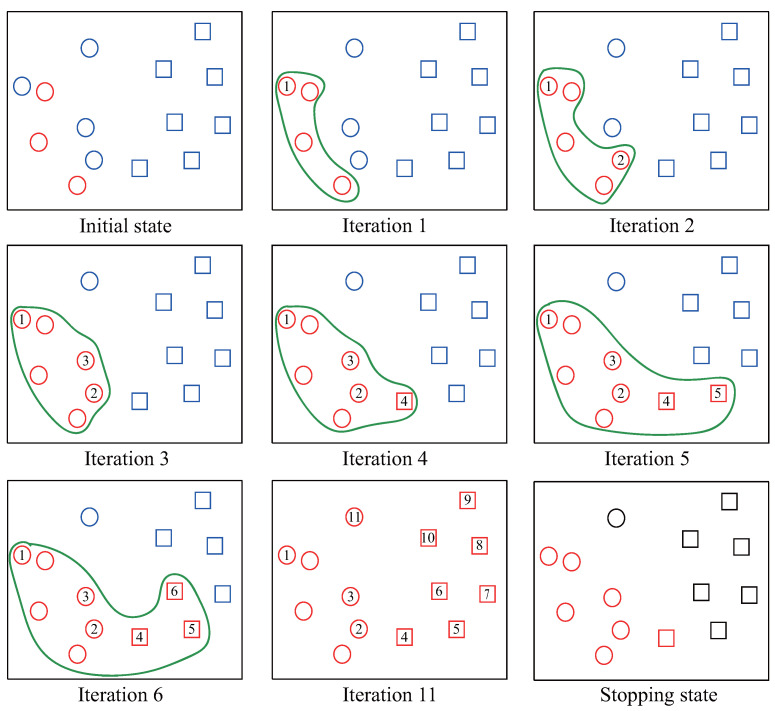
An illustration of *ST* procedure. In this example, the initial *PL* set has three instances.

In this subsection, we discuss how the *ST* algorithm works. The pseudo-code of the *ST* method for *PUTSC* can be found in Algorithm 1.
**Algorithm 1** The pseudo-code of the self-training method for *PUTSC*.**Require:**PL: Original positive labeled data; *U*: Unlabeled data.   **Ensure:**Positive: The time-series set labeled as positive.Negative: The time-series set labeled as negative.1:$list=\left\{\right\};i=1;info=\left\{\right\};PL^{\prime }=PL;$2:**while**U≠∅**do**3:  $x=\arg\min_{x\in U}\min_{x^{\prime }\in PL}\mathrm{Distance}\left(x,x^{\prime }\right)$;4:  list(i)=x;info(i)=Information(x,i);i=i+1;5:  U=U∖{x};PL=PL∪{x};6:**end while**7:stop=StoppingCriterion(info);8:$Positive=PL^{\prime }\cup \left\{list\left(1\right),list\left(2\right),\ldots ,list\left(stop\right)\right\}$;9:Negative={list(stop+1),list(stop+2),…,list(i)};10:**return**Positive,Negative;

The *ST* is an iterative method and it iteratively labels all time-series in *U* (line 2–6). In each iteration, *ST* first finds the unlabeled time-series *x* in *U* which is closest to *PL* based on the *one-nearest-neighbor (1NN)* formula (line 3). The **Distance()** is a function to measure the similarity between two time-series. Then, *ST* records the *x* and **Information(x,i)** in the map *list* and *info* in *i*-th iteration, respectively (line 4). The **Information()** is a function to record the important information of *x*. Note that the **Information()** is related to the stopping criterion **StoppingCriterion()**. For example, later we will see that when the stopping criterion confidence [8] is used, the **Information(x,i)** is the minimal distance between *x* and *PL*. Next, in line 5, the time-series *x* is removed from the *U* set and added into the *PL* set. After all time-series are labeled, the **StoppingCriterion()** estimates the number of positive time-series (line 7). The top *stop* time-series in *list* are labeled as positive (line 8) while the rest are labeled as negative (line 9). *ST* finally obtain the *Positive* set and *Negative* set which will be returned (line 10). We present a concrete example illustrated in Figure 4 to help reader gain an appreciation of the *ST*.

In Figure 4, at the initial state, the three red circles indicate the original *PL* set and all the blue instances indicate *U* set. In each iteration, the unlabeled instance which is closest to *PL* is labeled as positive and added to *PL*. For example, in iteration 1, the circle-1 is labeled because it is now closest to *PL*. We can observe that the labeled set *PL* is augmented gradually until all instances are labeled. After labelling all the instances in the set *U* (iteration 11), the *stopping criterion* calculates the stopping point to build the final positive set and negative set. Specifically, in our example, the value of *stop* is equal to 4 and at the stopping state, all red instances indicate the positive set while the rest are labeled as negative set.

### 2.3. Related Work for the Self-Training

From the Algorithm 1, we can observe that the similarity measure between two time-series (**Distance**) and the *stopping criterion* (**StoppingCriterion**) are two important parts of the *ST* method. The current researches for the *ST* method also focus on devising suitable similarity measures and stopping criteria. For similarity measure, representative similarity measures in the scope of *ST* include Euclidean Distance (ED) [7], Dynamic Time Warping (DTW) [8], DTW-D distance [13], and Maximum Diagonal Line of the Cross-Recurrence Quantification Analysis (MDL-CRQA) [14]. Note that the classic ED and DTW are two similarity measures that are widely used in the time-series mining community. As for stopping criterion, representative stopping methods include minimal nearest neighbor criterion [7], *stopping criterion* confidence (SCC) [8], Minimum Description Length principle based criterion [9,10,11], and Class Boundary Description by Graphic Analysis [12].

Our proposal is different from all the related works mentioned above in that: we focus on improving the performance of classic *ST* method by changing the *one-nearest-neighbor* selection formula (instruction 3 in Algorithm 1). We do not claim a contribution to the similarity measure or stop criterion used in *ST* method. Rather, we note that our proposal is orthogonal to the above works and can naturally be implemented along with state-of-the-art similarity measures or stop criteria (we will see an example in Section 3.4). In general, our contribution is an effective solution to the drawback of *ST* which may otherwise plague any attempt to *ST*.

## 3. Proposed Method

In this section, we first introduce the motivation behind our approach. Then, we describe two key techniques used in our method. We finally elaborate on our *ST-average* method and study the time complexity in detail.

### 3.1. Motivation

Despite the prevalence of the *ST*, we observe that it still has a drawback: the overall performance of *ST* may be sensitive to the initial labeled time-series. Usually, the probability to be mislabeled will be high when *PL* set includes the instances located near the boundary between the positive and negative classes. For example, in Figure 2a, we can see that *ST* incorrectly labels a negative data as positive class. This drawback motivates us to devise a more reliable and robust method.

In this paper, we propose the *ST-average* approach to overcome the drawback of conventional *ST*. Unlike *ST*, the proposed *ST-average* first calculates the average sequence of *PL* set and then finds the time-series *x* in *U* which is closest to average sequence. *ST-average* eliminates the effects of instances located near the negative and positive boundary by using the average sequence. Compared with any individuals in *PL* set, the average sequence may be more representative. A concrete example can be found in Figure 2b.

The key to *ST-average* algorithm is to calculate the average sequence of the *PL* set. However, it is not a trivial task to define the average sequence. The classical data can be averaged by their *mean* value using the traditional point-by-point averaging approach, but time-series cannot because they always vary in length and shifted in time axis (temporal aberrations) [22,23,24]. In the field of time-series data mining, the DTW distance is a well-established and widely used technique to address both issues, which can cope with shifted time-series of different lengths by looking for optimal alignment between two time-series. Thus, in *ST-average*, the average sequence is obtained in the DTW distance space. Since our method utilizes DTW technique and its averaging method, we first describe them in the next subsection.

### 3.2. Dynamic Time Warping

**Figure 5:**
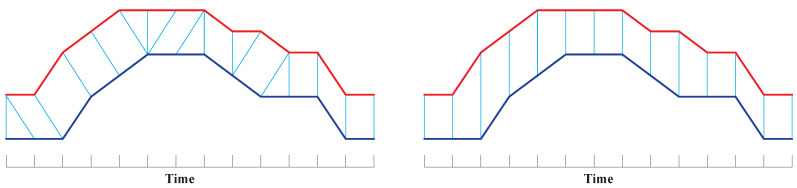
An optimal alignment generated by DTW (**left**), and a strictly time-rigid alignment generated by Euclidean Distance (**right**).

DTW [17,25] is the most popular distance in time-series, and performs well in similarity search because it allows time-flexible alignment and can find the best matching between two time-series by searching for *optimal alignments*; hence, it allows time-series to be locally shifted along the temporal axis and can handle time-series of unequal lengths. Figure 5 illustrates that compared with Euclidean Distance, DTW guarantees to obtain the optimal alignment between two time-series A=(1,1,2,2.5,3,3,3,3,2.5,2.5,2,2,1,1) and B=(1,1,1,2,2.5,3,3,3,2.5,2,2,2,1,1).

In DTW, an alignment from time-series $A=(a_{1},a_{2},\ldots ,a_{m})$ to time-series $B=(b_{1},b_{2},\ldots ,b_{n})$ is described by a *warping path*: $W=(w_{1},w_{2},\ldots ,w_{L})$, where *L* is the length of the warping path. The $k^{th}$ element $w_{k}=\left(i,j\right)$ represents the alignment from $a_{i}$ in *A* to $b_{j}$ in *B*. The warping path must satisfy the following constraints [26,27].

- *Boundary constraint*: $w_{1}=\left(1,1\right)$ and $w_{L}=\left(m,n\right)$.
- *Continuity-monotonically constraint*: $w_{k}-w_{k-1}\in \left\{\left(1,1\right),\left(0,1\right),\left(1,0\right)\right\}$.

Given a warping path *W*, the distance cost between *A* and *B* under *W* is calculated as follows:

(1) $$Dis_{W}\left(A,B\right)=\sum_{i=1}^{L}d\left(a_{w_{i}\left[1\right]},b_{w_{i}\left[2\right]}\right),$$

where $d\left(x,y\right)={\left(x-y\right)}^{2}$. Suppose that $W^{*}$ denotes the set of all warping paths, then the DTW distance between *A* and *B* is defined as follows:

(2) $$\mathrm{DTW}\left(A,B\right)=\min_{W\in W^{*}}Dis_{W}\left(A,B\right).$$

The *optimal alignment* from *A* to *B* is

(3) $$W^{OPT}\left(A,B\right)=\arg\min_{W}Dis_{W}\left(A,B\right).$$

Note that brute-force examining all possible warping paths to calculate the DTW distance could be very expensive or even infeasible because there are exponentially many possible warping paths. Fortunately, the DTW can be calculated by dynamic programming. Specifically, we can calculate DTW distance between *A* and *B* as follows.

(4) D(0,0)=0;D(0,1:n)=+∞;D(1:m,0)=+∞

(5) $$D\left(i,j\right)=d\left(a_{i},b_{j}\right)+min\left\{\begin{array}{c} D(i,j-1)\\ D(i-1,j)\\ D(i-1,j-1) \end{array}\right.$$

(6) DTW(A,B)=D(m,n).

The *D* is called the accumulated cost matrix and the optimal warping path can be found by back-tracking matrix *D* [24]. In this way, the time complexity of calculating DTW is O(mn).

### 3.3. Time-Series Averaging

Suppose $T=\{T_{1},T_{2},\ldots ,T_{N}\}$ is a set of *N* time-series. Generally, the time-series can have different lengths. The average sequence of *T* under the DTW distance can be formulated as follows [18,24].

(7) $$\bar{T}=\arg\min\frac{1}{N}\sum_{i=1}^{N}\mathrm{DTW}\left(T_{i},A\right),\forall A\in S^{l},\forall l\in \left[1,+\infty \right),$$

where $S^{l}$ indicates the space of all time-series of length *l*. We have to consider all possible values for length *l* because the information on the average sequence length is not available.

Many effective algorithms have been proposed to solve the time-series averaging problem [18,23,24,28,29]. Among these existing time-series averaging algorithms, the DTW barycenter averaging (DBA) [18] is the most cited solution up to date (more than 510 citations) and is widely used as the basis of many time-series applications such as clustering and classification [15]. Thus, in this paper, DBA is used as our averaging method and we briefly introduce how DBA works.

The DBA is an iterative method and it iteratively refines the initial selected randomly average sequence to obtain the final average sequence. In each iteration, the element $\bar{T}\left(k\right)$ in average sequence $\bar{T}$ is updated by averaging the elements in $T_{i}\left(i=1,2,\ldots ,N\right)$ which align with $\bar{T}\left(k\right)$ based on DTW. We provide the pseudo-code of DBA (Algorithm 2) to help reader reproduce our proposed method.
**Algorithm 2** The pseudo-code of the DBA method for averaging time-series set.**Require:**$T=\{T_{1},T_{2},\ldots ,T_{N}\}$: the time-series set to average.$\bar{T}$: initial average sequence (length *l*) selected from *T* randomly.IT: number of iterations.   **Ensure:**$\bar{T}$: the average sequence.1:**for**i=1:IT**do**2:  alignedSet(k)=∅,k=1,…,l;3:  **for** j=1:N **do**4:    Using DTW to align $\bar{T}$ and $T_{j}$;5:    **for** k=1:l6:      Identifying the elements $C_{k}$ in $T_{j}$ which align with element $\bar{T}\left(k\right)$;7:      $alignedSet\left(k\right)=alignedSet\left(k\right)\cup C_{k}$;8:    **end for**9:  **end for**10: **for** k=1:l **do**11:    $\bar{T}\left(k\right)=mean\left(alignedSet\left(k\right)\right)$; 12: **end for**13:**end for**14:**return** $\bar{T}$;

### 3.4. The ST-Average Method

**Figure 6:**
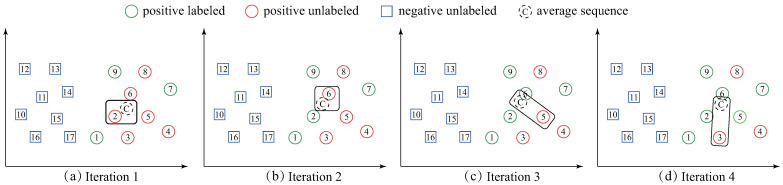
An illustration of the first four iterations of the *ST-average*. In each iteration, *ST-average* first computes average sequence *C* and then labels the time-series which is closest to *C*.

In this subsection, we present a formal and detailed description of the *ST-average* method. The pseudo-code can be found in Algorithm 3. It can be seen from Algorithm 3 that *ST-average* is based on the similar process of *ST* but adds the DBA technique (line 3). Besides, the unlabeled time-series which is closest to the average sequence is labeled (line 4). Figure 6 visualizes *ST-average*.
**Algorithm 3** The pseudo-code of the *ST-average* method for *PUTSC*.**Require:** PL: Original positive labeled data; *U*: Unlabeled data.**Ensure:** Positive: The time-series set labeled as positive. Negative: The time-series set labeled as negative.1:$list=\left\{\right\};i=1;info=\left\{\right\};PL^{\prime }=PL;$2:**while**U≠∅**do**3: center=DBA(PL);4: $x=\arg\min_{x\in U}\mathrm{Distance}\left(x,center\right)$;5: list(i)=x;info(i)=Information(x,i);i=i+1;6: U=U∖{x};PL=PL∪{x};7:**end while**8:stop=StoppingCriterion(info);9:$Positive=PL^{\prime }\cup \left\{list\left(1\right),list\left(2\right),\ldots ,list\left(stop\right)\right\}$;10:Negative={list(stop+1),list(stop+2),…,list(i)};11:**return**Positive,Negative;

Note that in Algorithm 3, the three functions **Distance**(), **Information**() and **StoppingCriterion**() are implicit. When using *ST-average* in practice, these three functions need to be implemented by explicit algorithms. For example, the **Distance**() can be simply implemented by DTW distance, Euclidean Distance or DTW-D distance and the **StoppingCriterion**() can be implemented by minimal nearest neighbor criterion [7], stopping criterion confidence [8] or Minimum Description Length principle based criterion [9,10,11].

For concreteness, in this part, we describe an explicit implementation of *ST-average* method which will be used in our experiments. Specifically, the function **Distance**() is implemented by DTW distance due to its effectiveness and popularity in the time-series domain. The **StoppingCriterion**() is implemented by *stopping criterion* confidence (*SCC*) [8], which is an improvement method of the seminal *stopping criterion* proposed in [7]. The *SCC* is defined as follows.

(8) $$SCC\left(i\right)=\frac{\left|info(i)-info(i-1)\right|}{Std(info(1),\ldots ,info(i))}\times \frac{\left|U|-(i-1\right)}{\left|U\right|},$$

in which |U| is the number of initial unlabeled time-series, Std is a standard deviation calculation function, and info(i) denotes the minimum DTW distance between the selected time-series *x* in iteration *i* and the closest time-series from PL set. That is,

(9) $$info\left(i\right)=\mathrm{Information}\left(x,i\right)=\min_{x^{\prime }\in PL}\mathrm{DTW}\left(x,x^{\prime }\right).$$

When using *SCC* criterion, the value of *stop* (line 8 in Algorithm 3) is calculated as follows (In literature [8], $stop={\arg\max}_{i\in [1,|U|]}SCC\left(i\right)-2.$ However, we find that when combined with our method, $stop={\arg\max}_{i\in [1,|U|]}SCC\left(i\right)-1$ can achieve better performance in our experiments.):

(10) $$stop=\mathrm{StoppingCriterion}\left(info\right)=\underset{i\in [1,|U|]}{\arg\max}SCC\left(i\right)-1.$$

We here just simply use the *SCC* criterion and we direct the reader to [8] for a comprehensive survey of *SCC*.

### 3.5. Time Complexity Analysis

This subsection shows the time complexity of *ST-average*. The *ST-average* process can be divided into four parts: (1) Computing DBA; (2) Finding the selected time-series *x* in iteration *i*. (3) Recording the information of *x*. (4) Calculating the stopping point. In this part, we assume that the similarity measure between time-series is implemented by DTW distance and all time-series have the same length *L*; thus, the complexity of computing DTW is $O(L^{2})$. Suppose that |PL| and |U| are the number of *initial* labeled time-series and unlabeled time-series, respectively, and *N* is the sum of |PL| and |U|, and *I* denotes the number of iterations in each DBA computation. From [18] we know that the DBA has a time complexity of $O(IKL^{2})$ to compute the average sequence from the *K* time-series. Thus, we can obtain the computation of DBA in *ST-average* requires

(11) $$\begin{array}{cc} C(DBA) & =O(I|PL|L^{2}+I\left(|PL|+1\right)L^{2}+\cdots +INL^{2})\\ \; & =O(IN^{2}L^{2}). \end{array}$$

In each iteration, *ST-average* must scan all the data in positive labeled set at that time to find the selected time-series *x*. Thus,

(12) $$\begin{array}{cc} C(find\_x) & =O(|PL|L^{2}+\left(|PL|+1\right)L^{2}+\cdots +NL^{2})\\ \; & =O(N^{2}L^{2}). \end{array}$$

The functions **information**() and **StoppingCriterion**() typically require time complexity that linearly with the *L* and |U|; thus, they are relatively minor and can be ignored in our analysis. Therefore, the overall *S-average* requires

(13) $$\begin{array}{cc} C_{ST-average} & =C(DBA)+C(find\_x)\\ \; & =O(\left(I+1\right)N^{2}L^{2}). \end{array}$$

As for *ST*, to improve its efficiency, we can store a look-up table which contains all of the DTW distance between pairs of time-series. Then, the distance DTW $\left(x,x^{\prime }\right)$ (line 3 in Algorithm 1) can be read from this look-up table. In this way,

(14) $$C_{ST}\left(find\_x\right)=O\left(N^{2}L^{2}\right).$$

Like *ST-average*, the time complexity of **information**() and **StoppingCriterion**() in *ST* can also be ignored. Thus, the conventional *ST* requires

(15) $$C_{ST}=O\left(N^{2}L^{2}\right).$$

Therefore, the time complexity of *ST* is smaller than our method *ST-average*. The speedup obtained by *ST* is

(16) $$Speedup=\frac{C_{ST-average}}{C_{ST}}=I+1.$$

## 4. Experimental Evaluation

### 4.1. Experimental Setup

#### 4.1.1. Algorithms

In this evaluation, four representative methods are used to illustrate the effectiveness of *ST-average* and we give a short introduction of each of them.

- *ST-SCC* is proposed in [8], and is one of the state-of-the-art algorithms for *PUTSC* problem, which uses DTW distance as the similarity measure and SCC as the stopping criterion.
- *C-MDL* is proposed in [9], which uses the constraint-based MDL principle for *PUTSC* problem. This method does not use any stopping criteria, but stops the self-training process when the number of time-series which does not satisfy the constraints exceeds the predefined threshold.
- *SCC-center-dtw* is our proposed method presented in Section 3.4, which utilizes the idea of *ST-average* and is an explicit implementation of *ST-average*.
- *SCC-center-ed* is similar to our *SCC-center-dtw* approach. The only difference is that *SCC-center-ed* uses the Euclidean Distance (ED) to calculate the *average sequence* while *SCC-center-dtw* uses DTW distance.

The above four algorithms are sufficient to show whether *ST-average* is effective. Specifically, from the comparison of *ST-SCC* and *SCC-center-dtw*, we can know whether the idea of *ST-average* can improve the performance of the original *ST-SCC*. From the comparison of *SCC-center-dtw* and *SCC-center-ed*, we can know whether it is necessary to calculate the *average sequence* under the DTW distance. Besides, the *C-MDL* and *SCC-center-dtw* can be regarded as two variants of *ST* technique, we are interested in which of these two variants performs better.

#### 4.1.2. The Performance Metric

We focus on evaluating the performance of labelling the original unlabeled instances (*U* set). This is equivalent to classifying the *U* set. The *F1-score* is used in our experiments to evaluate the performance of the proposal and baseline methods. *F1-score* is the harmonic mean of the recall (*r*) and precision (*p*), and can be defined as follows:

(17) $$F1=\frac{2\times p\times r}{p+r},$$

in which

(18) $$p=\frac{number\;\;of\;\;correct\;\;positive\;\;predictions}{number\;\;of\;\;positive\;\;predictions}$$

and

(19) $$r=\frac{number\;\;of\;\;correct\;\;positive\;\;predictions}{number\;\;of\;\;positive\;\;instances}.$$

From Equation (17) we know that the *F1-score* can be large only when both *r* and *p* are excellent. Therefore, *F1-score* is suitable for our purpose of classifying positive and negative instances accurately. Too low *p* or too low *r* is unacceptable, and can be reflected by a small value of *F1-score*.

To provide a more intuitive comparison of the performances among different approaches, we sort the labelling results [30]. The best-performing method ranks 1. If multiple approaches have the same *F1-score*, we set their rankings as the average of their corresponding rankings. For example, if *ST-SCC* and *SCC-center-ed* have same labelling results on the ECG5000 dataset, their rankings are supposed to be 1 and 2, respectively; therefore, we set their rankings as (1+2)/2=1.5. In addition, the standard deviation of the ranking is used to illustrate the performance difference of the method on different datasets.

#### 4.1.3. Datasets

In this study, 10 time-series datasets collected from the UCR Archive are used to evaluate the performance of all algorithms. The selected datasets vary highly in their time-series length, classes, number of positive samples and application fields. Detailed information about these widely used datasets can be found in Table 1.

The reader should note that the goal of our experiments is to perform the classification only for *U* set provided by training data; thus, unlike traditional machine learning tasks, our experiments do not involve any testing data. We aim to label the data in *U* as positive or negative. However, some of the datasets have more than two classes. Therefore, we select the data whose *class label = 1* as positive class, and all others as negative classes.

#### 4.1.4. Implementation Details

We implemented all algorithms in MATLAB (version R2019b), and ran all the experiments using Windows 10 enterprise with 2.30 GHz CPU (i7-9750H) and 16GB memory. We set the size of initial *PL* set |PL|=3 and the number of iterations in DBA I=15. In order to see the effects of the initial positive instances on the performance and eliminate the randomness, for each dataset, we repeat the experiments 20 times with various randomly selected initial positive instances.

In our implementation of DBA, the initial average sequence is selected randomly. We find that this will affect the *F1* value. To eliminate the impact of randomness, we run each of the 20 sets of randomly selected initial positive instances 20 times and calculate the mean and standard deviation of these 400 *F1* values. For other algorithms, since there is no uncertainty like DBA does, we only calculated the mean and standard deviation of the *F1* values of 20 sets of initial positive instances for each data set.

As for *C-MDL*, we set the predefined threshold to 5 according to [9] and the cardinality used in the MDL principle to 16.

### 4.2. F1-Score

This part presents the performance of labelling the *U* set. Table 2 reports the mean and standard deviation of *F1* values of each algorithm on different datasets. To compare the advantages and disadvantages of different algorithms more intuitively, we rank the mean of the *F1* value among different algorithms on the same dataset, as Table 3 illustrates. *SCC-center-dtw* attains the highest average ranking in the labelling the *U* set tasks. It proves that *SCC-center-dtw* performs well overall. Under the smallest standard deviation of ranking, the ranking of *SCC-center-dtw* is quite stable compared with other methods. It demonstrates that *SCC-center-dtw* is more effective and robust on different datasets than other methods.

In Table 3, it is apparent that *SCC-center-dtw* performs better than *ST-SCC* except in the *FiftyWords* dataset. It demonstrates that our *ST-average* idea can improve the performance of original *ST-SCC* method. From Table 2, we can find that *SCC-center-dtw* and *ST-SCC* perform similarly on the *FiftyWords* dataset. The mean F1 value of *ST-SCC* is 0.780, while it is 0.759 of *SCC-center-dtw*. Table 3 also shows that there are eight datasets on which *SCC-center-dtw* outperforms *SCC-center-ed*, which illustrates the necessity of using the DTW distance to average time-series. Besides, we can find that *SCC-center-dtw* performs better than *C-MDL* except in the *ItalyPowerDemand* and *ECG5000* dataset. The mean F1 value of *SCC-center-dtw* and *C-MDL* on the *ECG5000* dataset are 0.758 and 0.796 separately, and they are 0.603 and 0.684 when these two algorithms run on the *ItalyPowerDemand* dataset. Their labelling performance on these datasets is similar.

**Figure 7:**
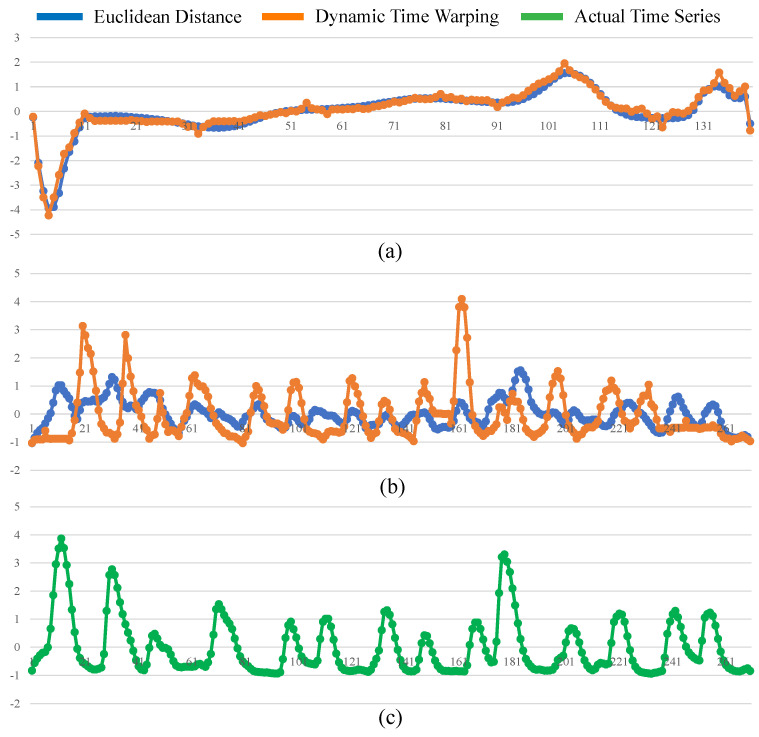
Average sequences obtained by Euclidean Distance and DTW distance in *ECG5000* dataset (**a**) and *FiftyWords* dataset (**b**). (**c**) The actual time-series in the *FiftyWords* dataset.

Note that, in some rare cases, the *SCC-center-dtw* and *SCC-center-ed* have the similar mean *F1* value. For example, in *ECG5000* dataset, the average *F1* value of *SCC-center-dtw* is 0.758 while is 0.891 for *SCC-center-ed*. It is because *SCC-center-dtw* and *SCC-center-ed* can obtain similar resultant average sequence. Figure 7a presents two average sequences calculated by Euclidean Distance and DTW distance in *ECG5000* dataset, respectively. It is obvious that these two sequences have the similar shape. In such case, there is no significant different between Euclidean Distance and DTW in averaging time-series. Therefore, *SCC-center-dtw* and *SCC-center-ed* have the similar performance in *ECG5000*. However, in *FiftyWords* dataset, averaged sequences shown in Figure 7b are totally different. Figure 7c shows the actual time-series in *FiftyWords* dataset. Obviously, this actual time-series has a similar shape to the average sequence calculated by DTW. Therefore, in *FiftyWords* dataset, *SCC-center-dtw* performs better than *SCC-center-ed*.

### 4.3. Running Time

**Figure 8:**
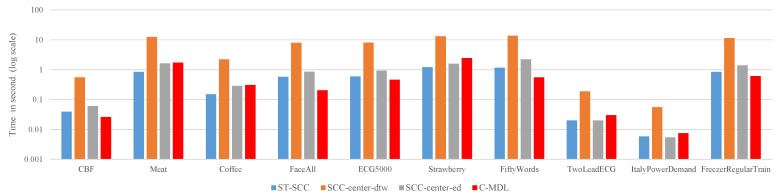
The efficiency of each method on different datasets. The *ST-SCC*, *SCC-center-ed* and *C-MDL* have similar performance. However, *SCC-center-dtw* is significantly more time-consuming than them.

Efficiency is another criteria for assessing algorithms. In this subsection, we evaluate the running time of four approaches. The average time required to label each unlabeled time-series is recorded and the results can be found in Figure 8. The results show that *SCC-center-dtw* is significantly more time-consuming than *ST-SCC*, *SCC-center-ed* and *C-MDL* on all datasets as we expected. We note that *ST-SCC* is 9.3 to 15 faster than *SCC-center-dtw*. In our experiments, the number of iterations in DBA I=15. Therefore, according to Equation (16), theoretically, the speedup obtained by *ST-SCC* is around 16. The results illustrated in Figure 8 generally confirm the time complexity analysis in Section 3.5.

Overall, compared to *ST-SCC*, *SCC-center-ed* and *C-MDL*, the *SCC-center-dtw* achieves better results with more running time. For some applications which are sensitive to the computational time, there exists some techniques to speed up *SCC-center-dtw*. First, the lower bound techniques [31] and the *PrunedDTW* [32] can be used to accelerate DTW computation. Second, the Sakoe-Chiba Band [33] or Itakura Parallelogram [34] can be utilized to speed up DBA procedure.

## 5. Conclusions and Future Work

In this paper, we propose the *ST-average* method to solve the *PUTSC* problem. Unlike traditional *ST*-based method, our proposal utilizes the average sequence of the *PL* set to decide which unlabeled time-series should be labeled and added into *PL* set. The average sequence is calculated by DBA technique, and we explain in detail the necessity of using DBA. We conduct extensive experiments on public datasets to demonstrate the efficiency and effectiveness of the proposed method. Experimental results show that our proposal achieves better results. The theoretical analysis and actual running time show that our method is slower than related approaches. We acknowledge this weakness and provide some feasible solutions.

It is worth noting that *ST-average* can possibly be combined with many similarity measures and *stopping criteria* used in classic *ST*-based method to provide even better results. In this paper, the *SCC-center-dtw* algorithm which utilizes DTW distance and SCC stopping criterion is presented. It is an interesting topic for future work to identify the best combination, and we might be able to use *active learning* technique to select the best combination, as literature [5] did. Another promising direction is examining another way to decide which unlabeled time-series should be labeled. For example, the time-series *x* which has the smallest sum of DTW distance from all instances in the *PL* can be labeled and added into *PL* set. It will be interesting to know what kind of selection method is the most effective.

## Figures and Tables

**Table 1:** Datasets descriptions.

No.	Dataset	Size of Training Data	Time-Series length	Classes	Number of Positive Samples	Type
1	CBF	30	128	3	10	Simulated
2	Meat	60	448	3	20	Spectro
3	Coffee	28	286	2	14	Spectro
4	FaceAll	560	131	14	40	Image
5	ECG5000	500	140	5	292	ECG
6	Strawberry	370	235	2	132	Spectro
7	FiftyWords	450	270	50	52	Image
8	TwoleadECG	23	82	2	12	ECG
9	ItalyPowerDemand	67	24	2	34	Sensor
10	FreezerRegularTrain	150	301	2	75	Sensor

**Table 2:** The mean(μ) and standard deviation(σ) of *F1* value of four methods on various datasets.

Dataset	ST-SCC μ±σ	SCC-Center-dtw μ±σ	SCC-Center-ed μ±σ	C-MDL μ±σ
CBF	0.669 ± 0.123	0.805 ± 0.101	0.771 ± 0.103	0.488 ± 0.175
Meat	0.550 ± 0.096	0.561 ± 0.083	0.580 ± 0.066	0.460 ± 0
Coffee	0.608 ± 0.200	0.814 ± 0.145	0.735 ± 0.210	0.611 ± 0
FaceAll	0.508 ± 0.144	0.552 ± 0.201	0.353 ± 0.073	0.469 ± 0.192
ECG5000	0.551 ± 0.087	0.758 ± 0.234	0.891 ± 0.015	0.796 ± 0.150
Strawberry	0.547 ± 0.088	0.615 ± 0.118	0.546 ± 0.129	0.520 ± 0
FiftyWords	0.780 ± 0.140	0.759 ± 0.135	0.063 ± 0.020	0.409 ± 0.206
TwoleadECG	0.563 ± 0.169	0.657 ± 0.174	0.464 ± 0.176	0.628 ± 0.076
ItalyPowerDemand	0.431 ± 0.147	0.603 ± 0.183	0.469 ± 0.218	0.684 ± 0.039
FreezerRegularTrain	0.285 ± 0.107	0.708 ± 0.046	0.702 ± 0.097	0.591 ± 0.131

**Table 3:** Ranking of different methods for the labelling performance for *U* set.

Dataset	*ST*-SCC	SCC-Center-dtw	SCC-Center-ed	C-MDL
CBF	3	1	2	4
Meat	3	2	1	4
Coffee	4	1	2	3
FaceAll	2	1	4	3
ECG5000	4	3	1	2
Strawberry	2	1	3	4
FiftyWords	1	2	4	3
TwoleadECG	3	1	4	2
ItalyPowerDemand	4	2	3	1
FreezerRegularTrain	4	1	2	3
Average ranking	3.0 ± 1.0	1.5 ± 0.67	2.6 ± 1.11	2.9 ± 0.94

## Data Availability

UCR datasets: https://www.cs.ucr.edu/~eamonn/time_series_data_2018/.

## References

[1] Chen L.J., Ho Y.H., Hsieh H.H., Huang S.T., Lee H.C., Mahajan S. (2017). ADF: An anomaly detection framework for large-scale PM2. 5 sensing systems. IEEE Internet Things J..

[2] Norgaard S., Saeedi R., Gebremedhin A.H. (2019). Multi-Sensor Time-Series Classification for Activity Tracking Under Variable Length. IEEE Sens. J..

[3] Kiranyaz S., Ince T., Gabbouj M. (2015). Real-time patient-specific ECG classification by 1-D convolutional neural networks. IEEE Trans. Biomed. Eng..

[4] Chen W., Shi K. (2019). A deep learning framework for time series classification using Relative Position Matrix and Convolutional Neural Network. Neurocomputing.

[5] Liang S., Zhang Y., Ma J. Active Model Selection for Positive Unlabeled Time Series Classification. Proceedings of the 2020 IEEE 36th International Conference on Data Engineering (ICDE).

[6] Nguyen M.N., Li X.L., Ng S.K. Positive unlabeled learning for time series classification. Proceedings of the Twenty-Second International Joint Conference on Artificial Intelligence.

[7] Wei L., Keogh E. Semi-supervised time series classification. Proceedings of the 12th ACM SIGKDD International Conference on Knowledge Discovery and Data Mining.

[8] Ratanamahatana C.A., Wanichsan D. (2008). Stopping criterion selection for efficient semi-supervised time series classification. Software Engineering, Artificial Intelligence, Networking and Parallel/Distributed Computing.

[9] Vinh V.T., Anh D.T. Constraint-based MDL principle for semi-supervised classification of time series. Proceedings of the 2015 Seventh International Conference on Knowledge and Systems Engineering (KSE).

[10] Begum N., Hu B., Rakthanmanon T., Keogh E. Towards a minimum description length based stopping criterion for semi-supervised time series classification. Proceedings of the 2013 IEEE 14th International Conference on Information Reuse & Integration (IRI).

[11] Vinh V.T., Anh D.T. (2016). Two novel techniques to improve mdl-based semi-supervised classification of time series. Transactions on Computational Collective Intelligence XXV.

[12] González M., Bergmeir C., Triguero I., Rodríguez Y., Benítez J.M. (2016). On the stopping criteria for k-nearest neighbor in positive unlabeled time series classification problems. Inf. Sci..

[13] Chen Y., Hu B., Keogh E., Batista G.E. DTW-D: Time series semi-supervised learning from a single example. Proceedings of the 19th ACM SIGKDD International Conference on Knowledge Discovery and Data Mining.

[14] de Carvalho Pagliosa L., de Mello R.F. (2018). Semi-supervised time series classification on positive and unlabeled problems using cross-recurrence quantification analysis. Pattern Recognit..

[15] Petitjean F., Forestier G., Webb G.I., Nicholson A.E., Chen Y., Keogh E. (2016). Faster and more accurate classification of time series by exploiting a novel dynamic time warping averaging algorithm. Knowl. Inf. Syst..

[16] Dau H.A., Keogh E., Kamgar K., Yeh C.C.M., Zhu Y., Gharghabi S., Ratanamahatana C.A., Hu B., Begum N., Bagnall A. (2018). The UCR Time Series Classification Archive. https://www.cs.ucr.edu/~eamonn/time_series_data_2018/.

[17] Berndt D.J., Clifford J. (1994). Using Dynamic Time Warping to Find Patterns in Time Series.

[18] Petitjean F., Ketterlin A., Gançarski P. (2011). A global averaging method for dynamic time warping, with applications to clustering. Pattern Recognit..

[19] Zhu X.J. (2005). Semi-Supervised Learning Literature Survey.

[20] Zhu X., Goldberg A.B. (2009). Introduction to semi-supervised learning. Synth. Lect. Artif. Intell. Mach. Learn..

[21] Nguyen M.N., Li X.L., Ng S.K. (2012). Ensemble based positive unlabeled learning for time series classification. International Conference on Database Systems for Advanced Applications.

[22] Niennattrakul V., Srisai D., Ratanamahatana C.A. (2012). Shape-based template matching for time series data. Knowl.-Based Syst..

[23] Morel M., Achard C., Kulpa R., Dubuisson S. (2018). Time-series averaging using constrained dynamic time warping with tolerance. Pattern Recognit..

[24] Liu Y.T., Zhang Y.A., Zeng M. (2019). Adaptive global time sequence averaging method using dynamic time warping. IEEE Trans. Signal Process..

[25] Rakthanmanon T., Campana B., Mueen A., Batista G., Westover B., Zhu Q., Zakaria J., Keogh E. Searching and mining trillions of time series subsequences under dynamic time warping. Proceedings of the 18th ACM SIGKDD International Conference on Knowledge Discovery and Data Mining.

[26] Keogh E.J., Pazzani M.J. Derivative dynamic time warping. Proceedings of the 2001 SIAM International Conference on Data Mining.

[27] Candan K.S., Rossini R., Wang X., Sapino M.L. (2012). sDTW: Computing DTW distances using locally relevant constraints based on salient feature alignments. Proc. VLDB Endow..

[28] Niennattrakul V., Ratanamahatana C.A. On clustering multimedia time series data using k-means and dynamic time warping. Proceedings of the 2007 International Conference on Multimedia and Ubiquitous Engineering (MUE’07).

[29] Leon-Alcaide P., Rodriguez-Benitez L., Castillo-Herrera E., Moreno-Garcia J., Jimenez-Linares L. (2020). An evolutionary approach for efficient prototyping of large time series datasets. Inf. Sci..

[30] Zhang M., Pi D. (2017). A new time series representation model and corresponding similarity measure for fast and accurate similarity detection. IEEE Access.

[31] Tan C.W., Petitjean F., Webb G.I. Elastic bands across the path: A new framework and method to lower bound DTW. Proceedings of the 2019 SIAM International Conference on Data Mining.

[32] Silva D.F., Batista G.E. Speeding up all-pairwise dynamic time warping matrix calculation. Proceedings of the 2016 SIAM International Conference on Data Mining.

[33] Sakoe H., Chiba S. (1978). Dynamic programming algorithm optimization for spoken word recognition. IEEE Trans. Acoust. Speech Signal Process..

[34] Itakura F. (1975). Minimum prediction residual principle applied to speech recognition. IEEE Trans. Acoust. Speech Signal Process..

